# DeepLMS: a deep learning predictive model for supporting online learning in the Covid-19 era

**DOI:** 10.1038/s41598-020-76740-9

**Published:** 2020-11-16

**Authors:** Sofia B. Dias, Sofia J. Hadjileontiadou, José Diniz, Leontios J. Hadjileontiadis

**Affiliations:** 1 CIPER, Faculdade de Motricidade Humana, Universidade de Lisboa, Lisbon, Portugal; 2 Department of Primary Education, Democritus University of Thrace, Alexandroupolis, Greece; 3 Department of Electrical Engineering and Computer Science, Khalifa University of Science and Technology, Abu Dhabi, UAE; 4 Healthcare Engineering Innovation Center, Department of Biomedical Engineering, Khalifa University of Science and Technology, Abu Dhabi, UAE; 5 Department of Electrical and Computer Engineering, Aristotle University of Thessaloniki, Thessaloníki, Greece

**Keywords:** Engineering, Mathematics and computing

## Abstract

Coronavirus (Covid-19) pandemic has imposed a complete shut-down of face-to-face teaching to universities and schools, forcing a crash course for online learning plans and technology for students and faculty. In the midst of this unprecedented crisis, video conferencing platforms (e.g., Zoom, WebEx, MS Teams) and learning management systems (LMSs), like Moodle, Blackboard and Google Classroom, are being adopted and heavily used as online learning environments (OLEs). However, as such media solely provide the platform for e-interaction, effective methods that can be used to predict the learner’s behavior in the OLEs, which should be available as supportive tools to educators and metacognitive triggers to learners. Here we show, for the first time, that Deep Learning techniques can be used to handle LMS users’ interaction data and form a novel predictive model, namely DeepLMS, that can forecast the quality of interaction (QoI) with LMS. Using Long Short-Term Memory (LSTM) networks, DeepLMS results in average testing Root Mean Square Error (RMSE) $<0.009$, and average correlation coefficient between ground truth and predicted QoI values $r\ge 0.97$ $(p<0.05)$, when tested on QoI data from one database pre- and two ones during-Covid-19 pandemic. DeepLMS personalized QoI forecasting scaffolds user’s online learning engagement and provides educators with an evaluation path, additionally to the content-related assessment, enriching the overall view on the learners’ motivation and participation in the learning process.

## Introduction

New designs of educational processes that include online learning have been flourishing in the last decades; some characteristic examples^1–3^ include affective (a-), blended (b-), collaborative (c-), mobile (m-), game (g-), transformative (t-), Cloud (Cl-), and ubiquitous (u-) learning, among others. Online learning improves access to education and training, aiming at reducing temporal and spatial problems that can be met in the traditional form of education^4,5^. In parallel, online learning has become one of the fastest growing industries, with a market growth rate over 900% since 2000, which is expected to reach in 2025 an impressive total market value of $325 billion^6^. Furthermore, as to the production and provision of online learning courses, the latter, when compared against the conventional Face-to-Face (F2F) ones, have an average consumption of 90% less energy and 85% fewer CO2 emissions produced per student^7^.

Online learning, though, asks for the combination of different delivery methodologies to contribute towards the optimization not only of the learning development, but also of deployment costs and time^8^. In this context, a key-factor that adds value to the quality of the learning experience is the quality of interaction (QoI) within an online learning environment (OLE). Apparently, effective integration of technology is needed^9^ to support QoI within OLEs. Hence, efficient blending of strategic decisions, adequate available resources, and quick thinking in implementation are necessary for the development of efficient online learning. Nowadays, this becomes more visible, when the worldwide emergency of the pandemic Coronavirus disease (Covid-19) impacts approximately 600 million learners across the Globe (https://en.unesco.org/covid19/educationresponse, accessed 19/10/2020), rigorously shifting traditional F2F teaching/learning to online one^10^.

Learning Management Systems (LMSs) frame a digital learning environment where the user’s learning behavior and it’s evaluation need to be efficiently amended^11^. LMSs (e.g., Moodle, https://moodle.org/) are actually embedded within OLE, which usually offer quick access, huge data management and a variety of Web-based tools^12,13^. As Herrington et al.^14^ state, the degree of interactions that take place within an educational context of reference is an essential predictor of its success. The QoI of the learner, within the LMS, is a strong efficacy indicator of the design and its ability to sustain online learning communities^15–18^. In particular, designed procedures within the learning environment can activate and sustain interactions towards learning. Then, upon these interactions^19^, knowledge can be extracted concerning the student’s preferred learning patterns while interacting with leaning resources, and/or while collaborating in groups. In this respect, empirical findings suggest that the commitment to the course workflow^20^, the connection time^21^ and the total number of accesses to the system^22^ are very important. Moreover, LMS-based online records can also be used to map individual- and population-level social jet lag, showing the potentiality of the LMS to provide behavioral information in terms of learning and attention deficits^23^, or emergence of novel relationships between social structure and performance^24^. From an educational data mining and learning analytics perspective, LMS was used to provide user track data within the online learning context, which were used as additional sources of information in: i) early detection of at-risk students on distance learning modules^25,26^, ii) findings as to learning dispositions^27,28^, iii) learning success and performance prediction^29–39^, and iv) learner behavior and goal attainment in Massive Open Online Courses (MOOCs) prediction^40^. Nonetheless, no evaluation of the QoI was performed, beyond merely descriptive statistics of the users’ interactions and their relation with the users’ performance, which were the main focus of the analysis.

The aforementioned place the need on the analysis of user’s LMS-based interactions related with their quality, so the latter could be used to explain the true nature of the users’ behavior when interacting within a LMS. So far, relevant research focused on QoI tends to examine LMS data statistics, including learner-teacher discussions and exchanges in online forums, to investigate the dimension, depth and category of interactions occurred^41^. A more extended and quantitative approach in QoI analysis was introduced by Dias and Diniz^42^. Their model, namely FuzzyQoI, considers the users’ (professors’ and students’) interactions, based on LMS use, and, by translating the knowledge of the experts in the field to fuzzy constructs, quantitatively estimates, a normalized index of the users’ QoI. As a result, the latter, can be used to identify users’ LMS interaction trends and provide personalised feedback to users. Another approach to evaluate the human interaction processes on a LMS-based online learning course was proposed by Dzandu and Tang^43^. They used a semiotic framework as guide to identify syntactic, semantic, pragmatic and social context gaps or problems, focusing on only the human information interaction issues. Nevertheless, their approach was based on simple questionnaires, missing out the dynamic characteristics of LMS interactions. In an further effort, Dias et al.^44^ suggested the use of a Fuzzy Cognitive Map (FCM) as a means to efficiently model the way LMS users interact with it, by estimating their QoI within a b-learning context. Their FCM-QoI model was used to analyse the QoI influential concepts’ contribution to self-sustained cycles (static analysis) and corresponding alterations, when the use of the LMS time period is considered (dynamic analysis), demonstrating potential to increase the flexibility and adaptivity of the QoI modeling and feedback approaches. In the work of Cerezo et al.^45^, identical students’ LMS Moodle logs behaviours were grouped concerning effort, time spent working, and procrastination, in order to investigate the students’ asynchronous learning processes, matching their behaviours with different achievement levels. Although this approach tries to shed light upon the role of the LMS interaction in the students’ achievements, it lacks generalisation power and evaluates the LMS-based QoI mainly from the grading of the students’ achievements and not from the actual interaction quality per se.

**Figure 1:**
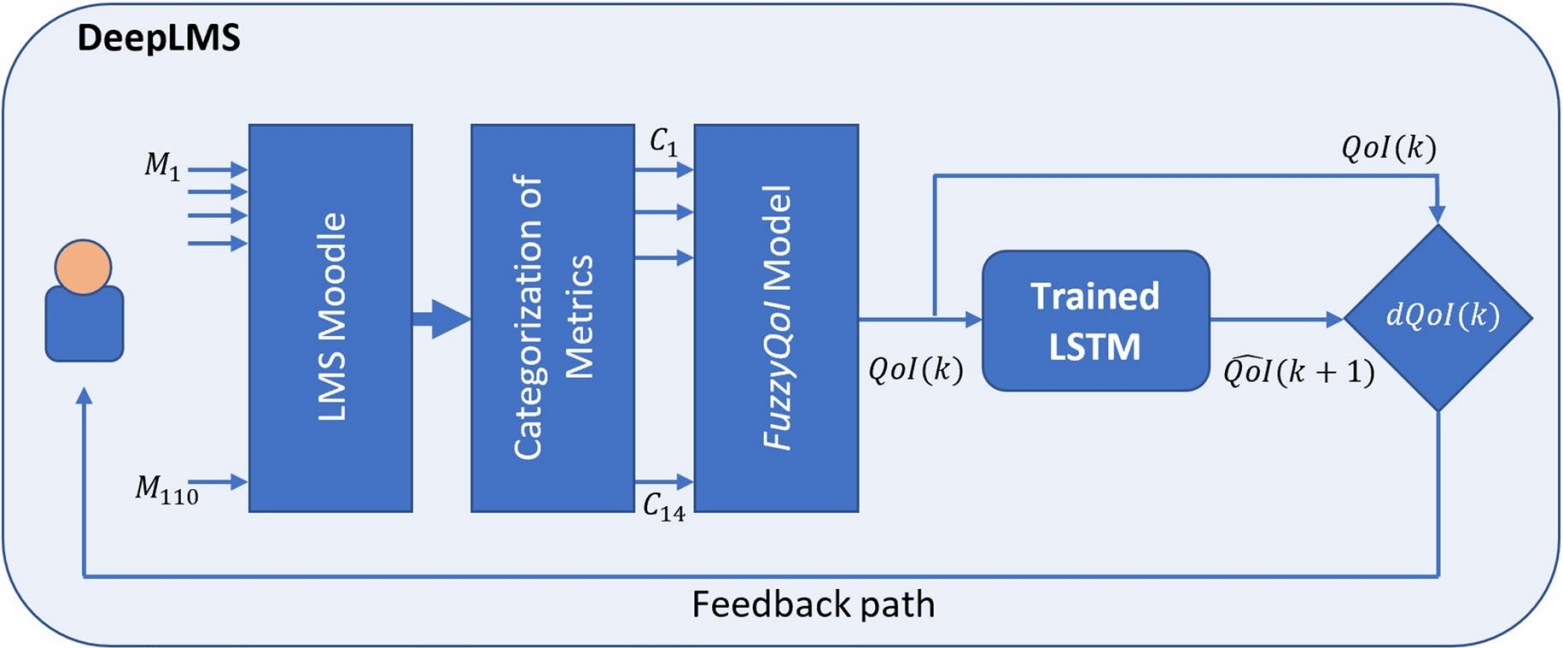
The DeepLMS-based QoI prediction concept. A schematic representation of the proposed DeepLMS functionality, with the LMS Moodle user’s interaction metrics ($M_1,\ldots , M_{110}$; see Supplementary Table S1) categorized into 14 input parameters ($C_1,\ldots , C_{14}$; see Supplementary Table S1) fed to the FuzzyQoI model^42^, outputting the estimated *QoI*(*k*) at instance *k*. The latter is then inputted to the trained LMST network (see “Methods” section) to predict the ${\hat{QoI}}(k+1)$ at instance $(k+1)$. Both *QoI*(*k*) and ${\hat{QoI}}(k+1)$ are compared and their difference (*dQoI*(*k*)) is used to inform the user’s feedback path.

The current work explores, for the first time, the predictive power that can be drawn from the analysis of the LMS-based QoI using Deep Learning. The proposed enhancement of LMS, namely DeepLMS, fills the gap in predictive use of LMS-based QoI to early inform effective feedback providers, i.e., educators, policy makers, relevant stakeholders, so to apply any corrective measure to increase the efficiency of the educational processes. In addition, DeepLMS acts as a metacognitive triggering tool to the learners, as it provides them with a prediction of their LMS-based QoI, so to reflect on their current QoI and proceed with any necessary personal corrective actions. By adopting a Long Short-Term Memory (LSTM) artificial Recurrent Neural Network (RNN) architecture^46^, a LSTM-based predictor was employed to form the QoI prediction model of DeepLMS, trained and tested on experimental LMS-based QoI data drawn from three databases, i.e., DB1^42^, DB2, and DB3, that come from both the pre- (DB1) and during- (DB2, DB3) Covid-19 pandemic periods, and refer to different countries, sociocultural and educational settings. The derived experimental results across DB1-DB3 show efficient predictive performance of the DeepLMS to accurately predict the daily QoI values, despite any temporal and/or educational setting differences. An illustration of the DeepLMS-based QoI prediction process is depicted in Fig. 1.

## Results

As the three examined databases come from different countries (Portugal, United Arab Emirates, Greece), and refer to different time periods, i.e., pre- (DB1) and during-Covid-19 pandemic (DB2, DB3), and systemic settings, i.e., macro: Higher-Educational Institution (HEI)’s level (DB1), meso: course level (DB2), and micro: focused discipline level (DB3), the performance of the proposed DeepLMS approach is separately presented per database.

### DB1-related performance

**Figure 2:**
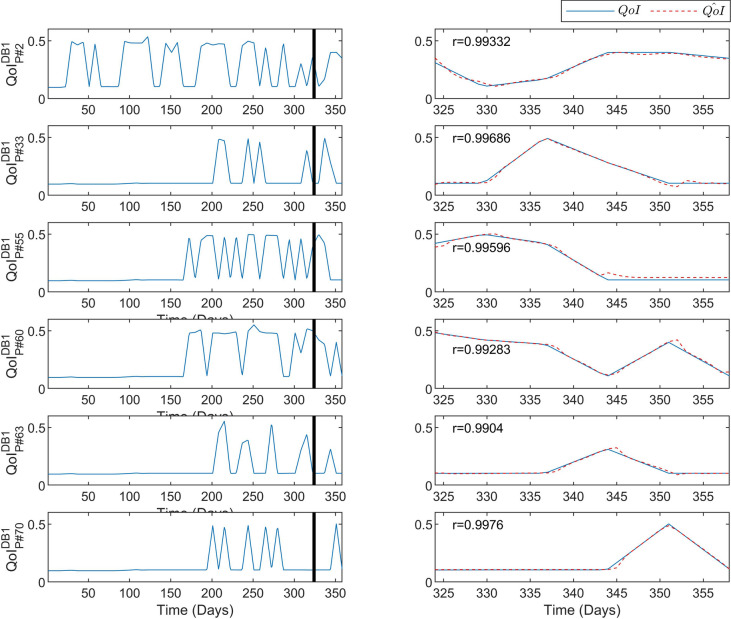
Predictive performance of the DeepLMS on QoI time series from DB1 Professors. The left column shows the QoI data from DB1 P#2, P#33, P#35, P#60, P#65, and P#70, used for training (from day 1 until day 323 where the vertical solid line lies) and for testing (day 324 until day 358), whereas the right column zooms into the testing QoI data (blue solid line) and the DeepLMS predicted QoI (red dashed line). Moreover, the estimated correlation coefficient *r* between the testing and the estimated QoI data (see “Methods” section) for each case is also superimposed in the right column plots.

Figure 2 depicts the predictive performance of the DeepLMS upon some excerpts of the QoI time series derived from the DB1 75 Professors (P#2, P#33, P#35, P#60, P#65, and P#70). In particular, the left column of Fig. 2 shows the QoI data used for training (from day 1 until day 323 where the vertical solid line lies) and for testing (day 324 until the day 358), whereas the right column zooms into the testing QoI data (blue solid line) and the DeepLMS predicted QoI (red dashed line). Moreover, in the right column of Fig. 2, the estimated correlation coefficient *r* between the testing and the estimated QoI data (see “Methods” section) for each case is also superimposed. These cases of QoI were selected to showcase the predictive performance of DeepLMS on QoI time series that have various patterns across the whole duration of the two academic semesters (358 days). In particular, for the case of P#2, an almost periodic pattern is noticeable, where the alteration between $QoI=0.11$ and $QoI=0.5$ is visible. Nevertheless, this is not evident in the rest of the cases, where there are sparse alterations of QoI with various frequency alterations between $QoI=0.11$ and $QoI=0.5$ values. As the LSTM network is capable of forgetting data that are not useful for its predictive performance, the predictive results shown in the right column of Fig. 2 and the corresponding *r* values, justify the efficient predictive performance of the proposed DeepLMS approach. One step further, in the right column of Fig. 2 for the case of P#55, out of all of the changes in slope across the depicted subfigures, this is the only one that contains a proactive prediction value, i.e., not just reacting to the QoI change (blue solid line) with a slight lag due to the use of the recurrent LSTM model.

**Figure 3:**
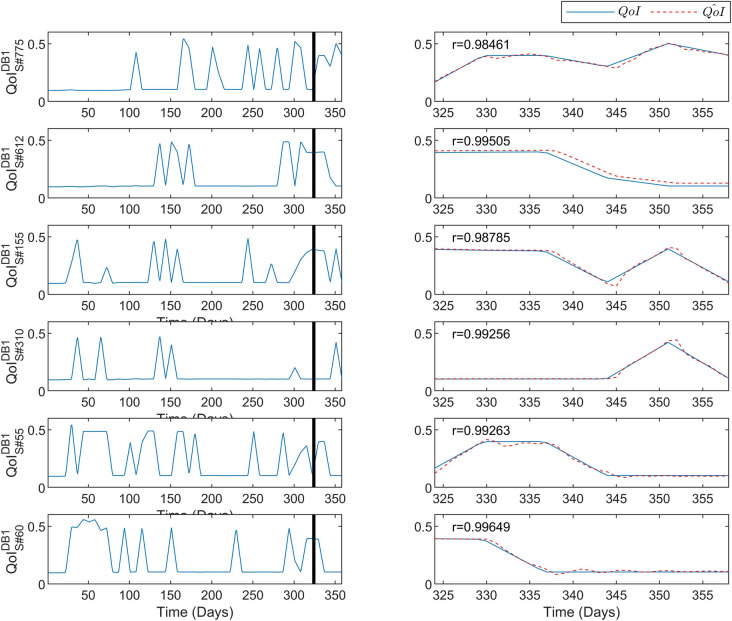
Predictive performance of the DeepLMS on QoI time series from DB1 Students. The left column shows the QoI data from DB1 S#55, S#60, S#155, S#310, S#612, and S#775, used for training (from day 1 until day 323 where the vertical solid line lies) and for testing (day 324 until day 358), whereas the right column zooms into the testing QoI data (blue solid line) and the DeepLMS predicted QoI (red dashed line). Moreover, the estimated correlation coefficient *r* between the testing and the estimated QoI data (see “Methods” section) for each case is also superimposed in the right column plots.

Similarly to Fig. 2, Fig. 3 depicts the predictive performance of the DeepLMS upon some excerpts of the QoI time series derived from the DB1 1037 Students (S#55, S#60, S#155, S#310, S#612, and S#775). The same configuration as in Fig. 2 is also followed in Fig. 3, where again various cases of the QoI time series distribution across the two academic semesters are shown. The solid line at day 323 separates the data used for the training from the ones used for testing. Moreover, the estimated correlation coefficient *r* per case is also depicted. From the results presented in Fig. 3, the same level of high predictive performance of DeepLMS seen in Fig. 2 is sustained for the case of Students.

**Figure 4:**
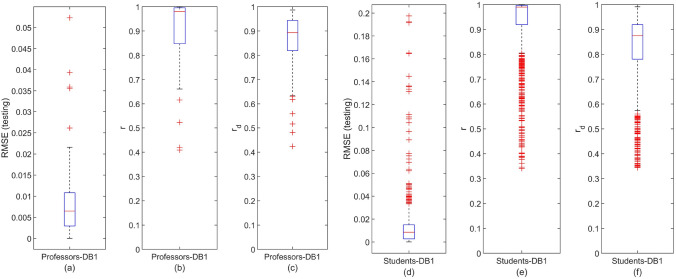
Distribution of the DeepLMS predictive performance indices across users’ groups of DB1. (**a**) Box-plot of the distribution of RMSE between the testing and the estimated QoI data for the case of DB1 Professors, (**b**) box-plot of the distribution of the correlation coefficient *r* between the testing and the estimated QoI data for the case of DB1 Professors, (**c**) box-plot of the distribution of the correlation coefficient $r_d$ between the derivative of the testing and the derivative of the estimated QoI data for the case of DB1 Professors; (**d**-**f**) same as (**a**–**c**), respectively, yet for the case of DB1 Students. Each box-plot visualises the interquartile range (height of rectangle), spanning the first (bottom) to the third quartile (top), the median value (horizontal red line inside the rectangle), the minimum and maximum values (ends of “whiskers” below and above the box, respectively) still within the interquartile range, and outlier values (individual red crosses below and above “whiskers”). Additional DeepLMS predictive performance indices are tabulated in Table 1.

Figure 4 illustrates the distribution of the DeepLMS performance indices (see “Methods” section) across the whole set of QoI data per user type (DB1 Professors (75): Fig. 4a–c; DB1 Students (1037): Fig. 4d–f. In particular, the distribution of the Root Mean Square Error (RMSE) between the testing and the estimated QoI data is depicted in Fig. 4a,d for the case of DB1 Professors and Students, respectively. Moreover, the distribution of the correlation coefficient *r* between the testing and the estimated QoI data is depicted in Fig. 4b,e for the case of DB1 Professors and Students, respectively. Furthermore, the distribution of the correlation coefficient $r_d$ between the derivative of the testing and the derivative of the estimated QoI data is depicted in Fig. 4c,f for the case of DB1 Professors and Students, respectively. The median and the 95% Confidence Interval (CI) of the estimated RMSE, *r* and $r_d$ are tabulated in Table 1. From both Fig. 4a,d and Table 1, it is clear that the testing RMSE lies in quite satisfactory levels across the two users’ groups of DB1, showing an efficient predictive performance by the DeepLMS. This is further justified by the very strong correlation identified both between the amplitude of the testing and predicted QoI values (Fig. 4b,e, Table 1) and the trend of the testing and predicted QoI values (Fig. 4c,f, Table 1). The number of outliers (red crosses in Fig. 4 lying outside > 1.5 times the interquartile range, i.e., the box-plot whiskers) does not really affect the overall predictive performance of the DeepLMS, as expressed by the corresponding high median values and low 95% CIs (Table 1). Moreover, the difference in the number of outliers between Fig. 4a–f comes from the distinct difference in the number of users per group (DB1 75 Professors vs. 1037 Students).

**Table 1:** DeepLMS and baseline FCM-QoI^44^ predictive performance indices across the whole set of QoI data per database and users’ group.

Database	Users’ Groups	DeepLMS			FCM-QoI^44^	
		Median$\pm 95$% *CI*			Median ± 95%CI	
	User Type	*RMSE*	*r*	$r_d$	*RMSE*	*r*
DB1	Professors (75)	0.0065 ± 0.0022	0.98 ± 0.06	0.87 ± 0.08	0.0360 ± 0.012	0.4045 ± 0.02
	Students (1037)	0.0086 ± 0.0012	0.99 ± 0.01	0.86 ± 0.02	0.0264 ± 0.003	0.5363 ± 0.01
DB2	Professors (3)	0.0043 ± 0.0095	0.96 ± 0.03	0.66 ± 0.34	N/A	N/A
	Students (180)	0.0038 ± 0.0046	0.94 ± 0.01	0.74 ± 0.04	N/A	N/A
DB3	Professor (1)	0.0172	0.99	0.90	N/A	N/A
	Students (52)	0.0039 ± 0.0098	0.99 ± 0.08	0.90 ± 0.09	N/A	N/A

The number in parenthesis denotes the number of members per users’ group; 95%*CI* denotes the 95% Confidence Interval; *RMSE* corresponds to the Root Mean Square Error, whereas *r* and $r_d$ (only for the DeepLMS case) correspond to the correlation coefficient between the testing and the estimated QoI data and between the derivative of the testing and the derivative of the estimated QoI data, respectively. N/A denotes the non applicable case, as the baseline FCM-QoI model was only applied to DB1^44^, which, though, has the highest number of LMS users. For the case of DB3-Professor, the *RMSE*, *r*, and $r_d$ values are provided, instead of the mean ± 95%CI ones, as there is only one Professor involved in DB3. Clearly, based on the estimated metrics, an efficient predictive performance of the proposed DeepLMS scheme is noticed, compared to the baseline FCM-QoI model^44^ predictive performance.

### DB2-related performance

**Figure 5:**
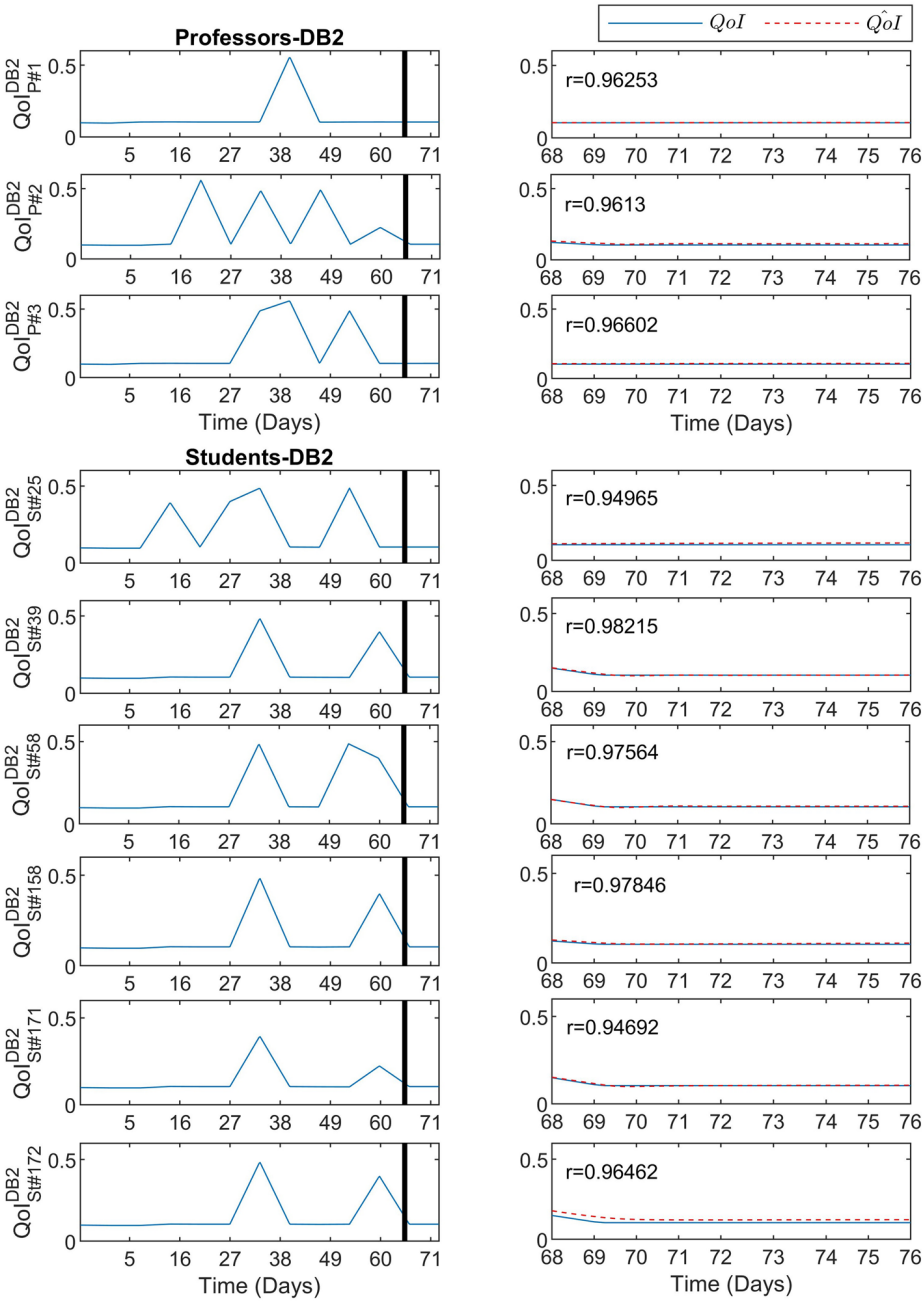
Predictive performance of the DeepLMS on QoI time series from DB2 Professors and Students. The left column-top panel shows the QoI data from the three DB2 Professors, i.e., P#1, P#2, and P#3, used for training (from day 1 until day 68 where the vertical solid line lies) and for testing (day 69 until day 76), whereas the left column-bottom panel shows the QoI data from excerpts of DB2 Students, i.e., S#25, S#39, S#58, S#158, S#171, and S#172, for the same training and testing periods. The right column (both panels) zooms into the testing QoI data (blue solid line) and the DeepLMS predicted QoI (red dashed line), including also the estimated correlation coefficient *r* between the testing and the estimated QoI data for each case.

Similarly to the results presented in the previous subsection for DB1, Fig. 5 depicts the predictive performance of the DeepLMS upon the QoI time series derived from the DB2 3 Professors (Fig. 5-top panel: P#1, P#2, and P#3) and from some excerpts from the DB2 180 Students (Fig. 5-bottom panel: S#25, S#39, S#58, S#158, S#171, and S#172). In both panels, the left column of Fig. 5 shows the QoI data used for training (before the vertical solid line) and for testing (after the vertical solid line), whereas the right column zooms into the testing QoI data (blue solid line) and the DeepLMS predicted QoI (red dashed line). Analogously to Figs. 2 and 3, the estimated correlation coefficient *r* between the testing and the estimated QoI data for each case is also superimposed on the right column of Fig. 5. As it can be seen from Fig. 5, the QoI time series involved in the testing part tends to converge to a constant value ($QoI=0.11$), whereas in the training QoI data there are notable alterations of QoI between $QoI=0.11$ and $QoI=0.5$ values. This difference in QoI values can potentially be attributed to the fact that the last days of the course were devoted to the demo presentation of students’ projects; hence, the focus was mostly placed on hands-on activities rather than LMS interactions. From the results depicted in Fig. 5, it is evident that the DeepLMS captures such change in the QoI values, exhibiting efficient performance in predicting the underlying trend.

**Figure 6:**
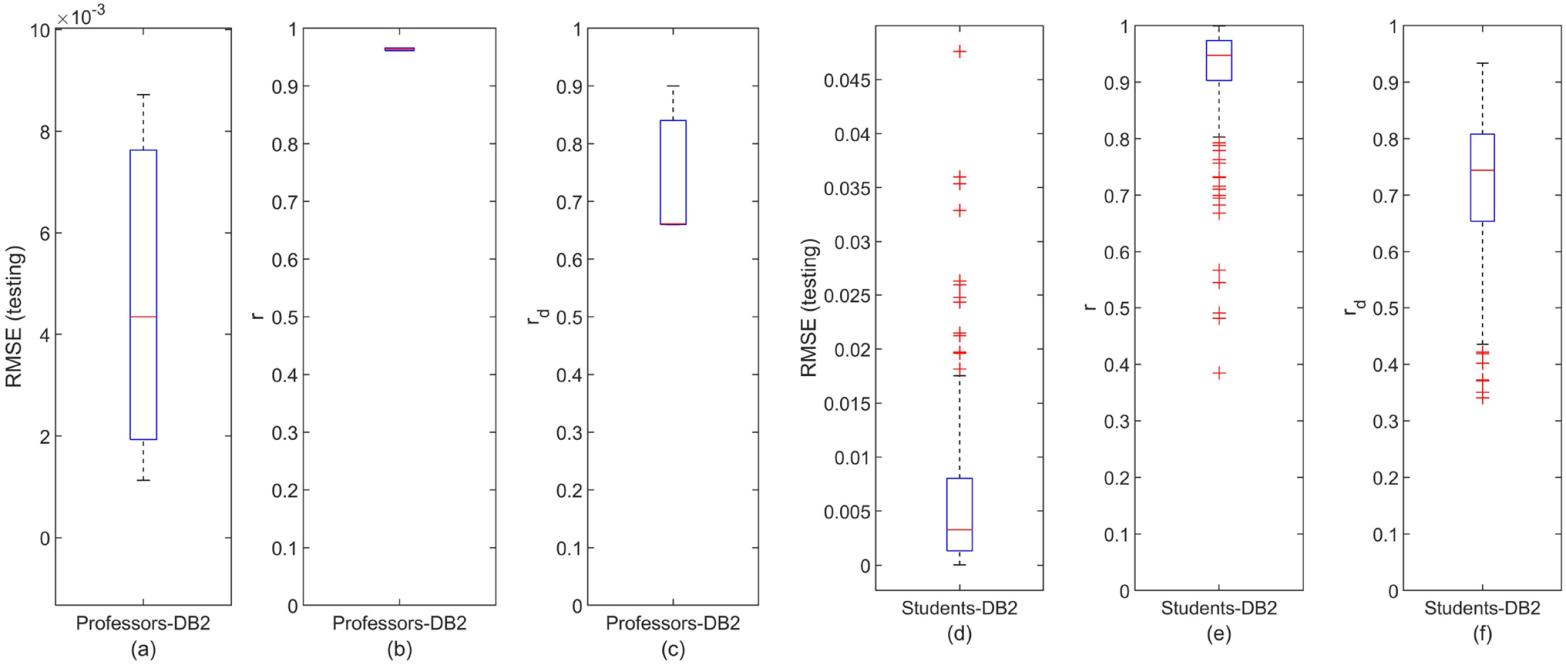
Distribution of the DeepLMS predictive performance indices across users’ groups of DB2. (**a**) Box-plot of the distribution of RMSE between the testing and the estimated QoI data for the case of DB2 Professors, (**b**) box-plot of the distribution of the correlation coefficient *r* between the testing and the estimated QoI data for the case of DB2 Professors, (**c**) box-plot of the distribution of the correlation coefficient $r_d$ between the derivative of the testing and the derivative of the estimated QoI data for the case of DB2 Professors; (**d**–**f**) same as (**a**–**c**), respectively, yet for the case of DB2 Students. Additional DeepLMS predictive performance indices are tabulated in Table 1.

Consonantly to Figs. 4 and 6 illustrates the distribution of the DeepLMS performance indices, i.e., RMSE, *r*, and $r_d$, across the whole set of QoI data per user type (DB2 Professors (3): Fig. 6a–c; DB2 Students (180): Fig. 6d–f). The corresponding values of the median and 95% CI of the estimated RMSE, *r*, and $r_d$ for the DeepLMS performance when using DB2 QoI data are tabulated in Table 1. From both Fig. 6 and Table 1, it is clear that DeepLMS sustains its efficient predictive performance reported in the case of DB1 also in the case of DB2, exhibiting quite satisfactory performance metrics across the two users’ groups of DB2. Apparently, the differences between DB1 and DB2 performance metrics are due to the different number of users per database (see Table 2); however, they are both quite acceptable.

**Table 2:** Characteristics of the databases used.

DB#	HEI (Country)	Time period start:end (days)	Covid-19	Scale	User type	No.	Sex male/female	Age range (mean ± std) (yrs)	LMS interactions
DB1	FMH (PT)	26/8/2019: 18/8/2010 (358)	Pre	HEI Level	Professors	75	36/39	24-54 (47.19 ± 8.8)	94,288
					Students	1037	466/571	18-48 (25.05 ± 5.9)	516,487
					Total	1112	502/610		610,775
DB2	KUST (UAE)	17/3/2020: 31/5/2020 (76)	During	Course Level	Professors	3	1/2	28-42 (33.60 ± 7.3)	1218
					Students	180	82/98	18-20 (18.36 ± 0.52)	8428
					Total	183	83/100		9646
DB3	AUTH (GR)	29/3/2020: 25/9/2020 (181)	During	Discipline Level	Professor	1	1/0	54	683
					Students	52	32/20	22-25 (23.23 ± 1.16)	26,373
					Total	53	33/20		27,056
					Grand Total	1348	618/730		647,477

DB: Database; HEI: Higher-Educational Institution; FMH: Faculdade de Motricidade Humana; KUST: Khalifa University of Science and Technology; AUTH: Aristotle University of Thessaloniki; PT: Portugal; UAE: United Arab Emirates; GR: Greece. The characteristics of the LMS users show balanced groups per sex, in both user types (Professor/Student), and almost a doubled mean age in the Professors compared to Students, as expected. Overall, more than 647,000 LMS interactions are considered in the construction of the related QoI values.

### DB3-related performance

**Figure 7:**
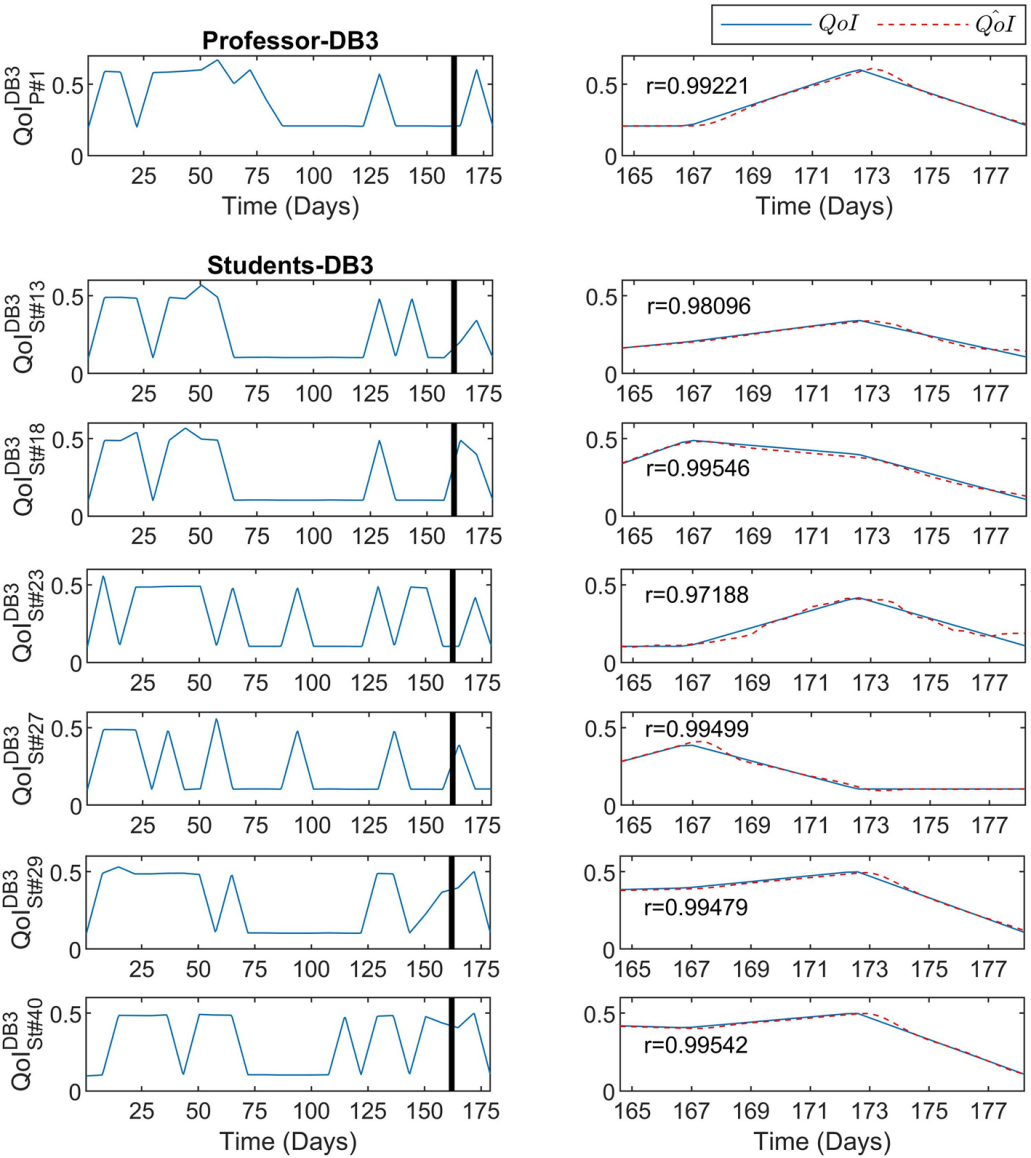
Predictive performance of the DeepLMS on QoI time series from DB3 Professor and Students. The left column-top panel shows the QoI data from the one DB3 Professor, i.e., P#1, used for training (from day 1 until day 163 where the vertical solid line lies) and for testing (day 164 until day 181), whereas the left column-bottom panel shows the QoI data from excerpts of DB3 Students, i.e., S#13, S#18, S#23, S#27, S#29, and S#40, for the same training and testing periods. The right column (both panels) zooms into the testing QoI data (blue solid line) and the DeepLMS predicted QoI (red dashed line), including also the estimated correlation coefficient *r* between the testing and the estimated QoI data for each case.

Figure 7 depicts the predictive performance of the DeepLMS upon the QoI time series derived from the DB3 1 Professor (Fig. 7-top panel: P#1) and from some excerpts from the DB3 52 Students (Fig. 7-bottom panel: S#13, S#18, S#23, S#27, S#29, and S#40), presented at the same format as in Fig. 5. As it can be seen from Fig. 7, the QoI time series involved in the testing part, unlike the ones depicted in Fig. 5, exhibit alterations between $QoI=0.11$ and $QoI=0.5$ values similar to the ones observed in the training QoI data, resembling also the patterns followed in Figs. 2 and 3. This can potentially be explained by the difference in the way the focused discipline related to DB3 was evaluated during the exams period, as it involves more online research-based activities, rather than the hands-on ones seen in DB2. From the results depicted in Fig. 7, it is clear that the DeepLMS takes into account the QoI characteristics from the training period and successfully predicts the various QoI patterns seen in the testing period.

**Figure 8:**
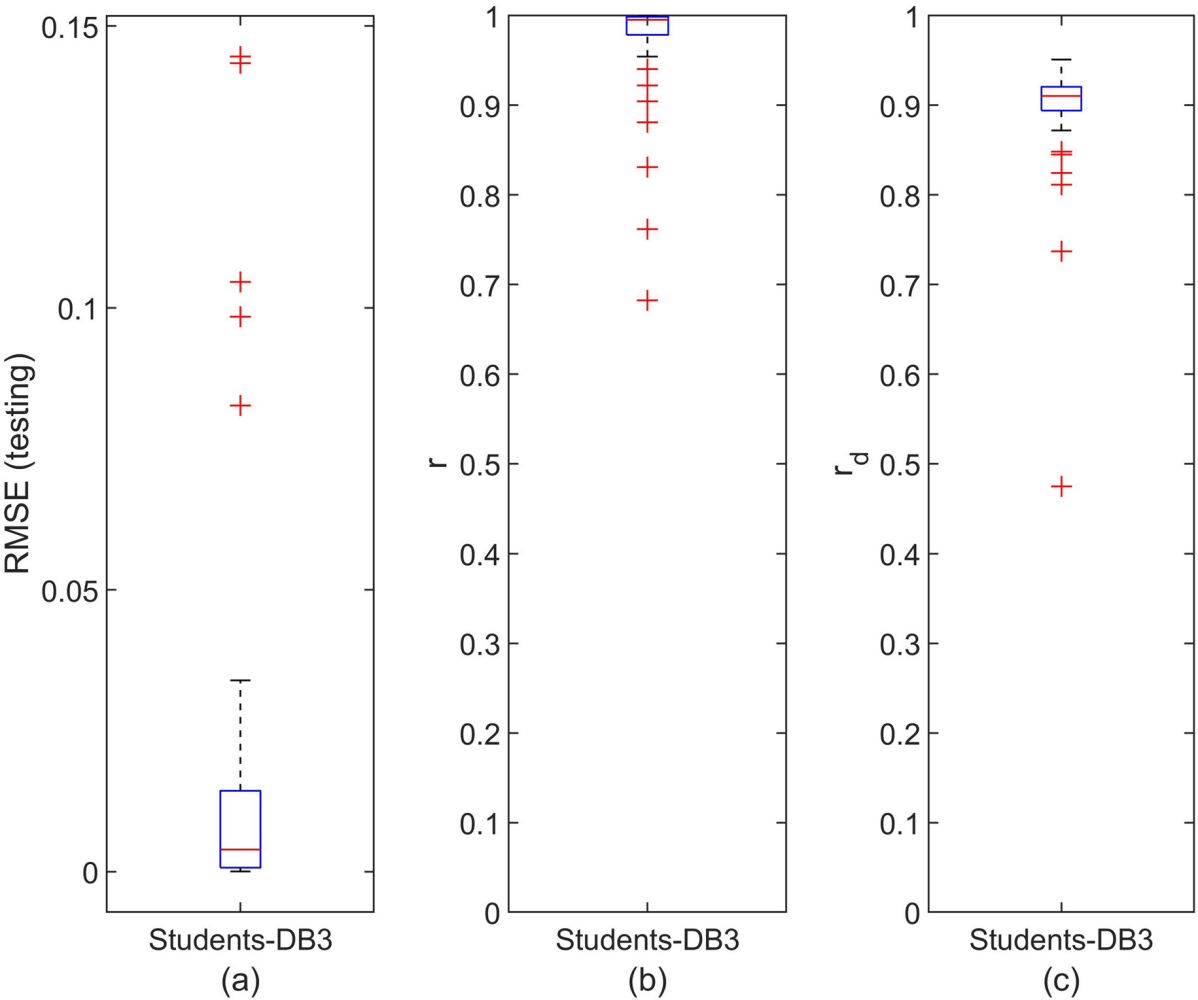
Distribution of the DeepLMS predictive performance indices across the DB3 Students. (**a**) Box-plot of the distribution of RMSE between the testing and the estimated QoI data for the case of DB3 Students, (**b**) box-plot of the distribution of the correlation coefficient *r* between the testing and the estimated QoI data for the case of DB3 Students, (**c**) box-plot of the distribution of the correlation coefficient $r_d$ between the derivative of the testing and the derivative of the estimated QoI data for the case of DB3 Students. Additional DeepLMS predictive performance indices are tabulated in Table 1.

The distribution of the DeepLMS performance indices, i.e., RMSE, *r*, and $r_d$, across the whole set of QoI data for the case of DB3 Students (52) is illustrated in Figs. 8a–c, respectively. The distribution for the case of DB3 Professors was omitted, as there is only one Professor involved within the DB3. The corresponding values of the median and 95% CI of the estimated RMSE, *r*, and $r_d$ for the DeepLMS performance when using DB3 QoI data are tabulated in Table 1. The results presented both in Fig. 8 and Table 1, confirm efficient predictive performance of the DeepLMS when using QoI data from DB3, similarly to the cases of DB1 and DB2. Apparently, the different number of users per database (see Table 2) contributes to the differences seen in the DeepLMS performance metrics across DB1, DB2 and DB3; yet, in all cases, the DeepLMS predictive performance can be considered quite satisfactorily.

## Discussion

In the unprecedented era of Covid-19, an alteration in the landscape for online education is clearly manifested by the hundreds of thousands of educators and learners setting out to academic cyberspace and OLEs. This is a paradigmatic change, a ‘black swan’ moment^47^, as the unforeseen event of Covid-19 pandemic ushers the educational practices in video conferencing platforms (e.g., Zoom, WebEx, MS Teams) and LMS-based uses. Surely, there is a high variability in the way educators act online (often for the first time) for offering remote instruction to their students outside the physical classroom. The abrupt ending of in-person classes leading to online settings can speed up the adoption of OLEs as learning mediators. Nevertheless, this instructional change, in such a compressed time frame, has the risk to solely create an insipid copy of today’s best online learning practices. Possibly, this is due, in part, to the lack of investment in online education modality by many educational institutions and/or to underestimation of online learning as a core aspect of their learner experience^48^. However, led by top universities, a noticeable change began a few years ago, as fully digital academic experiences started flourishing^49^. The current crisis due to Covid-19 is accelerating this trend, revealing the need for Higher Education Institutions (HEIs) to promote faculty’s digital skills. The latter can be facilitated by the construction of a technological backbone, to mitigate the effects of this crisis and to welcome the online teaching/learning within a digital era. These digital competences can amalgamate the short-term response to crisis into an enduring digital transformation of education contexts.

In this disrupted educational landscape, the issues of how instructors and colleges treat student evaluation and how institutions treat student evaluations of professors have surfaced. Educators face a challenge/opportunity in trying to evaluate quality, even as the educational activity they are evaluating is mutating, in real time. DeepLMS comes into foreground as a means to offer quantitative metrics of the user’s LMS-based QoI, as an alternative to conventional evaluation metrics. The efficient predictive performance of the DeepLMS, as justified by the experimental results derived from three databases, involving pre- and during-Covid-19 pandemic data, establishes a reliable basis to construct a motivational, personalized feedback to the LMS users, so to readjust their interaction with the LMS, as an effort to increase the related QoI. The latter refers to the efficient engagement of the user with the online part of the learning process (nowadays almost the sole one), and provides educators with an evaluation path, in parallel to the content-related assessment, that could enrich their overall view about learner motivation and participation in the learning process. Moreover, based on the estimated *dQoI*(*k*) and its segmentation setting (see “Methods” section), personalized feedback can be provided to users that helps them get the most out of their interaction with the LMS and the related online material, and can have a significant impact on overall learning performance outcomes^50^. Many forms of representation of the segmented *dQoI*(*k*) value can be employed (e.g., text, graphs, audiovisual); in all of them, however, a personalization in the way the feedback is communicated to users should be incorporated. For example, when $dQoI(k)\in [-1,-0.8]$, a text message of *‘Serious alert! Your QoI is expected to significantly fall!’*, can be used as an intense warning; alternatively, it can be more constructive in the form of *‘From now on, please try to be more focused and more active in the online part of your course’*. The former textual feedback is a descriptive interpretation of the *dQoI*(*k*) value *per se*, whereas the latter is a proactive interpretation that motivates learners to act constructively. This need for personalised interpretation stems from the fact that learners, usually, chose different paths to respond to learning challenges. For example, the ones with a positive orientation view feedback (either positive or negative) as information to be assimilated and accommodated. However, the ones with a negative orientation, perceive negative feedback as a ‘crushing blow’ and reflection of their poor ability^51^; most of such learners easily give up^50^. Hence, the adaptation of the feedback path could better support the ultimate aim in feedback provision, which allows learners to take over the function of assessing themselves and others^52^.

Within the aforementioned context, yet from a more integrated perspective, the proposed DeepLMS approach can be augmented to become an ideal mechanism/feedback to support various stakeholder groups in the domain of education (including department heads, teachers, administrators, technical support staff, and learners). This can be achieved by aggregating the individual predictive user outputs. This process could lead to effective technology-enabled learning. Amongst its attributes, it should include a focus on enduring learning outcomes. This endurance is reinforced by the DeepLMS through its focusing on the QoI prediction, whose dynamic feedback to LMS users, gradually etches in them the optimized LMS interaction as an enduring learning outcome.

From the results presented in Figs. 2, 3, 4, 5, 6, 7 and 8 and Table 1, the proposed DeepLMS seems independent of the group type, as it shows a similar predictive performance both in Professors’ and Students’ QoI prediction (Wilcoxon rank sum test for DB1: $p=0.070$). In addition, cross-country/scale/time-period statistical analysis has resulted in non-significantly statistical differences of the performance of DeepLMS for different sociocultural and temporal settings (Wilcoxon rank sum test for $RMSE(DB1,DB2): p=0.207; RMSE(DB1,DB3): p=0.219; RMSE(DB2,DB3)$: *p* = 0.387). The same holds for the factors of sex and age, as linear regression tests did not show any influence of both on the prediction of QoI for Professors ($\{sex, age\}_{P-DB1}: \{p=0.363, p=0.113\}$) and Students ($\{sex, age\}_{S-DB1}: \{p=0.415, p=0.167\}; \{sex, age\}_{S-DB2}: \{p=0.465, p=0.673\}; \{sex, age\}_{S-DB3}: \{p=0.508, p=0.693\}$). Note that the statistics related to Professors were estimated for DB1 only, as the number of Professors in DB2 (3) and DB3 (1) is limited. Moreover, DeepLMS seems insensitive to the sparsity of the interaction, as it efficiently models the user’s LMS-based various interaction patterns, as expressed in the QoI time-series morphology across time (Figs. 2, 3, 5, 7). These patterns are governed by various academic calendar activities, e.g., lectures, mid-term exams, final exams, winter/spring/summer breaks, and/or external ones, especially for DB2 and DB3, as the lockdown due to Covid-19 pandemic (DB2: 26/3-24/4/2020; DB3: 11/3-4/5/2020) lies within their time duration (see Table 2). In spite of these, the DeepLMS acknowledges such data evolution, resulting in adequate predictive performance due to the ability of its embedded LSTM forecasting model to outperform classical time series methods in cases with long, interdependent and sparse time series^53^. Clearly, the aforementioned results show increased generalization power in the performance of DeepLMS. Extending the demographics analysis in the bias domain, as Table 2 shows, the distribution of Male/Female was quite balanced, both in Professors and Students, along with their age, without any heterogeneity that would potentially produce data bias in LSTM learning. Hence, *no historical bias* (as no socio-technical issues were involved), *no representation bias* (sufficient number of users were involved and the significant spread of QoI data comes from users across three countries, with five courses with 30-40 different disciplines each course (macro level: DB1), one course (meso level: DB2) and one discipline (micro level: DB3)), *no measurement bias* (data were captured from users that all had equal access to the LMS Moodle pages after logged in), *no evaluation bias* (the evaluation was performed on an equal basis and with the same objective measures of (*RMSE*, *r*) as in the baseline algorithm (FCM-QoI^44^)), *no population bias* (user population represented in the datasets is coming from a real-life setting (University) end expresses the original target population), *no Simpson’s Paradox* (the data were homogeneous and there were no subgroups in Professors’ and Students’ groups), *no sampling bias* (uniform sampling across all groups), *no user-interaction bias* (the LMS Moodle metrics involved in the production of the QoI are 112 (see Table S1) and provide a high variety to the user to interact with the LMS Moodle), and *no self-selection bias* (the data were analyzed after the users interacted with the LMS and they were totally unaware of the research; hence, they could not influence the results by selective self-participation). Consequently, there is no unfairness arising from biases in the data. Taking the bias issue further, another source of unfairness could potentially arise from the learning algorithm involved itself. To avoid such event, some techniques^54^ could be explored to try to transform the data (pre-processing), so that the underlying discrimination is removed, or incorporating changes into the objective function or imposing a constraint (in-processing), or accessing a holdout set, which was not involved during the training of the model, and reassign the initially assigned labels by the model based on a function (post-processing). In the DeepLMS case, although no data bias was identified, in a broader perspective, flexibly fair representation in DeepLMS learning could be introduced in its future edition by creating a layer that disentangles the information that relate with sensitive attributes (e.g., demographics) and create a targeted learning for such sensitive latent variables, which potentially can bias the model, and incorporate such knowledge in a debias process (e.g., as in^55,56^) at the higher QoI prediction layer.

When performing a relevant comparative analysis between the DeepLMS performance and the most related FCM-QoI model^44^, that it is also based on the same QoI data drawn from the FuzzyQoI model^42^, it seems that the proposed DeepLMS achieves higher overall performance, when compared to the testing output of FCM-QoI. In particular, based on the predictive performance of both the DeepLMS and the FCM-QoI^44^ tabulated in Table 1, it is apparent that the DeepLMS exhibits lower testing RMSE and higher *r* values in its predictive output, when compared with the ones from the FCM-QoI model^44^. From a structural comparison, DeepLMS overcomes the training limitation of the FCM-QoI, i.e., its training is based on the mean values of QoI across users provided by the FuzzyQoI model; this, however, merges the specific characteristics of each user to an average behavior^44^. On the contrary, the DeepLMS provides personalised predictions for the QoI of each user across the academic semesters.

From a more general perspective, DeepLMS aligns with the previous efforts that incorporate LSTM-based predictions in the context of online education, yet not at the exact same specific problem settings as in DeepLMS. Hence, the latter is well-positioned with the approaches related to: i) cross-domains analysis, e.g., MOOCs impact in different contexts^57^, as DeepLMS could be easily adapted to a micro analysis of the QoI per discipline/course and transfer learning from one discipline to another at the same course (or courses with comparable content), as shown here with the application of DeepLMS to DB1-DB3, in a similar manner that was applied in MOOCs from different domains^57^; ii) combination of learning patterns in the context domain with the temporal nature of the clickstream data^58^, and identification of students at risk^59^, as DeepLMS could be combined with an auto-encoder to capture both the underlying behavioral patterns and the temporal nature of the interaction data at various levels of the predicted QoI (e.g., low (<0.5) QoI (at risk level)); iii) predicting learning gains by incorporating skills discovery^60,61^, as DeepLMS could provide the predicted QoI as an additional source of the user profile to his/her skills and learning gains; iv) user learning states and learning activities prediction from wearable devices^62^, as DeepLMS could easily be embedded in the expanded space of affective (a-) learning, and inform a more extended predictive model that would incorporate the learning state with the estimated QoI; v) increasing the communication of the instructional staff to learners based on individual predictions of their engagement during MOOCs^63,64^, as DeepLMS could facilitate the coordination of the instructor with the learner based on the informed predicted QoI; and vi) predicting the learning paths/performance^65^ and the teaching paths^66^, as the DeepLMS could be extended in the context of affecting the learning/teaching path by the predicted QoI.

Despite the promising results of the proposed DeepLMS towards prediction of the user’s LMS-based QoI, certain limitations exist. In particular, no correlation analysis with the content evaluation outcome from, e.g., quizzes, mid-/final exams, was undertaken. In fact, this was left for a future endeavor, as the focus here was to explore the predictive performance of the DeepLMS in LMS-based QoI prediction, fostering the role of the latter as an additional, to conventional grading, assessment field. Moreover, the data used here refer to one (2009/2010) or half (2020) academic year; thus, exploration of the DeepLMS application and further validation of its predictive performance upon follow-up data, i.e., monitoring of the same users across sequential academic years, would shed light upon the consistency in the predictive performance of the DeepLMS across longer time periods.

The efficient performance of the DeepLMS was validated on real data, incorporating adequate number of users and LMS data logs from different countries and educational settings. Since the structure and training of the proposed DeepLMS are not restricted to a specific course content, actually they were tested on human kinetics (DB1), engineering design (DB2), and advanced signal processing (DB3) educational contents, it could easily be expanded to the analysis of LMS data coming from various fields, e.g., Social Sciences, Medical and/or Engineering Education^67^. This would allow for the exploration of any dis/similarities and correlations in the LMS users’ QoI, from an institutional perspective. Moreover, as the Covid-19 pandemic shifted the use of LMS Moodle to Secondary Education Institutions (SEIs), as well, the DeepLMS could be used for comparing the LMS-based QoI across younger student groups and explore the age-related trends in LMS-based interaction.

As part of our future work on DeepLMS, we aim to perform a fusion of other measures of user’s quality in the online learning context at both SEIs and HEIs. This includes prediction of the Quality of Collaboration (QoC)^68^ and Quality of Affective Engagement (QoAE)^1,69^, in an effort to predict, in a holistic way, the various components that play significant role in the learning process, i.e., interaction, collaboration and affectiveness^1^. The incorporation of Deep Learning-based predictions of QoC and QoAE, in parallel to the QoI ones, extends the work of the authors^70–72^ from the concept of affective/blended/collaborative-teaching/learning (a/b/c-TEACH, http://abcteach.fmh.ulisboa.pt/) to the a/b/c/d(eep)-TEACH one. In the midst of the Covid-19 pandemic, such an AI-based scaffolding helps educators and learners move from quick fixes, and their possible consequence of regressing to poor practice, to maximum efficiency of the online learning tools available and truly support learning. Finally, as distinct time periods of pre-, during- and post-Covid-19 lockdown have been formed, the analysis of LMS data that emerged during these three periods seems promising, in particular for the identification of any effect on the QoI *per se* and its related prediction via the proposed DeepLMS model. This analysis will allow for further evaluation of the DeepLMS model predictive robustness against effects caused by time-related disruptors, such as Covid-19, in the context of education; ongoing efforts towards such direction are reported in^73^.

## Methods

The proposed DeepLMS approach explores the predictive power of Deep Learning in estimating the user’s LMS-based QoI within an online learning context, from his/her historical QoI data. This efficient QoI prediction feeds the feedback path (see Fig. 1), in an effort to provide metacognitive stimulus to learners and timely inform the educators as to their possible lack of motivation and course focus and/or adoption of unstructured online course interaction, alerting for preventive and corrective interventions. The performance of the DeepLMS was evaluated on QoI data estimated from $>647.000$ LMS Moodle interactions, as described below.

### Dataset

The LMS Moodle data used in DeepLMS were drawn from three databases, i.e., DB1, DB2 and DB3. The users’ characteristics and their contribution in LMS interactions per database, along with the related HEI, country, time period, Covid-19 association, and scale, are tabulated in Table 2. In particular, DB1 refers to the data included in the work of Dias and Diniz^42^, with 610,775 in total users’ LMS interactions, across two academic semesters (358 days) of the 2009/2010 academic year. All users started to use LMS Moodle in that academic year. These contributions were provided by the users (75 Professors and 1,037 Students) within five b-learning-based undergraduate courses, i.e., Sport Sciences, Ergonomics, Dance, Sport Management and Psychomotor Rehabilitation, offered by a public HEI (Faculdade de Motricidade Humana, Portugal). DB2 includes overall 9,646 users’ LMS online learning interactions drawn from Khalifa University of Science and Technology (KUST), Abu Dhabi, UAE, during the Spring semester of 2020 (76 days). These contributions were provided by the users (3 Professors and 180 Students) during the course of Engineering Design. The latter is a freshman course on the basic principles of engineering design, applied on solving real-life problems via projects. DB3 includes overall 27,056 users’ LMS online learning interactions drawn from a discipline in the area of Advanced Signal Processing at the Department of Electrical and Computer Engineering (ECE), Aristotle University of Thessaloniki (AUTH), Greece, taught at the 4th year of ECE studies, during the Spring semester and Summer/Fall exam periods of 2020 (181 days). The LMS contributions come from one Professor and 52 Students; the discipline is focused in techniques and algorithms of advanced signal processing, as a means to propose efficient solutions in research problems. The set of the available 110 LMS Moodle metrics ($M_1-M_{110}$ in Fig. 1; see Supplementary Table S1) logged by the users were corresponded to 14 categories ($C_1-C_{14}$ in Fig. 1; see Supplementary Table S1) that formed the inputs to the FuzzyQoI model^42^. The latter outputted the QoI estimations per user across the whole time-span of the analysis, which was kept the same across all databases, i.e., 358 days as in DB1, by using linear interpolation in the cases of DB2 and DB3; yet, displaying the initial length (DB1: 76 days; DB3: 180 days) in all resulted illustrations (Figs. 2, 3, 5, 7). These QoI daily estimations were used as ground-truth inputs to an LSTM-based predictor (Fig. 1) for training and testing (see relevant subsections below). More details of the QoI estimation from the FuzzyQoI model can be found in the work of Dias and Diniz^42^.

It should be noted that all data used here were de-identified (any information that would allow individual’s identity was stripped out). DB1 data come from the two authors’ (S.D and J.D) previous work^42^, where they had ethics clearance for research purpose use; hence, no ethical approval is needed for their reuse here. The use of DB2 data was approved by the KUST Ethics Committee (Protocol #: H20-021, 17.6.2020), whereas access to DB3 for research purpose use was granted by the AUTH eLearning Administrator to the last author (L.H), who was the responsible Professor of the related discipline.

### DeepLMS predictive performance evaluation

The predictive performance of the DeepLMS was separately evaluated for the two user types, i.e., Professors and Students (Table 2), analyzing their testing data in terms of: (a) the RMSE between the QoI values from the FuzzyQoI model^42^, i.e., $QoI^{FuzzyQoI}$, and the ones predicted by the DeepLMS, i.e., $QoI^{DeepLMS}$, (b) the correlation coefficient *r* between the $QoI^{FuzzyQoI}$ and $QoI^{DeepLMS}$, in order to evaluate the correctness in the estimation of the $QoI^{FuzzyQoI}$ values, and (c) the correlation coefficient $r_d$ between the derivative of the $QoI^{FuzzyQoI}$ and the derivative of the $QoI^{DeepLMS}$, in order to evaluate the correctness in the estimation of the $QoI^{FuzzyQoI}$ dynamics trend (increase/decrease). In both *r* and $r_d$ estimations, the value of $p\le 0.05$ was used for adopting them as statistically significant. Finally, the distributions of (a)-(c) across the whole set per user type were estimated (displayed as boxplots), in order to evaluate the overall predictive performance of the proposed DeepLMS approach.

### User’s feedback path triggering

For the triggering of the user’s feedback path (Fig. 1), the difference, at instance *k*, between the $$QoI^{FuzzyQoI}(k)$$ and $${\hat{QoI}}^{DeepLMS}(k+1)$$ is estimated, i.e., $$dQoI(k)={\hat{QoI}}^{DeepLMS}(k+1)-QoI^{FuzzyQoI}(k)$$, considering the use of an already trained LSTM network. As all estimated QoI values are normalized within [0,1], the estimated *dQoI*(*k*) ranges between $$[-1,1]$$. Positive *dQoI*(*k*) values can be used for a rewarding user’s feedback, whereas negative *dQoI*(*k*) values can be used for a warning one. Segmentation of the *dQoI*(*k*) range [−1,1] to different subsets, e.g., $$[-1,-0.8), [-0.8,-0.5), [-0.5,-0.3), [-0.3,-0.1) [-0.1,0.1), [0.1,0.3), [0.3,0.5), [0.5,0.8)$$ and [0.8, 1], could allow for flexibility in the granularity of the feedback construction.

**Figure 9:**
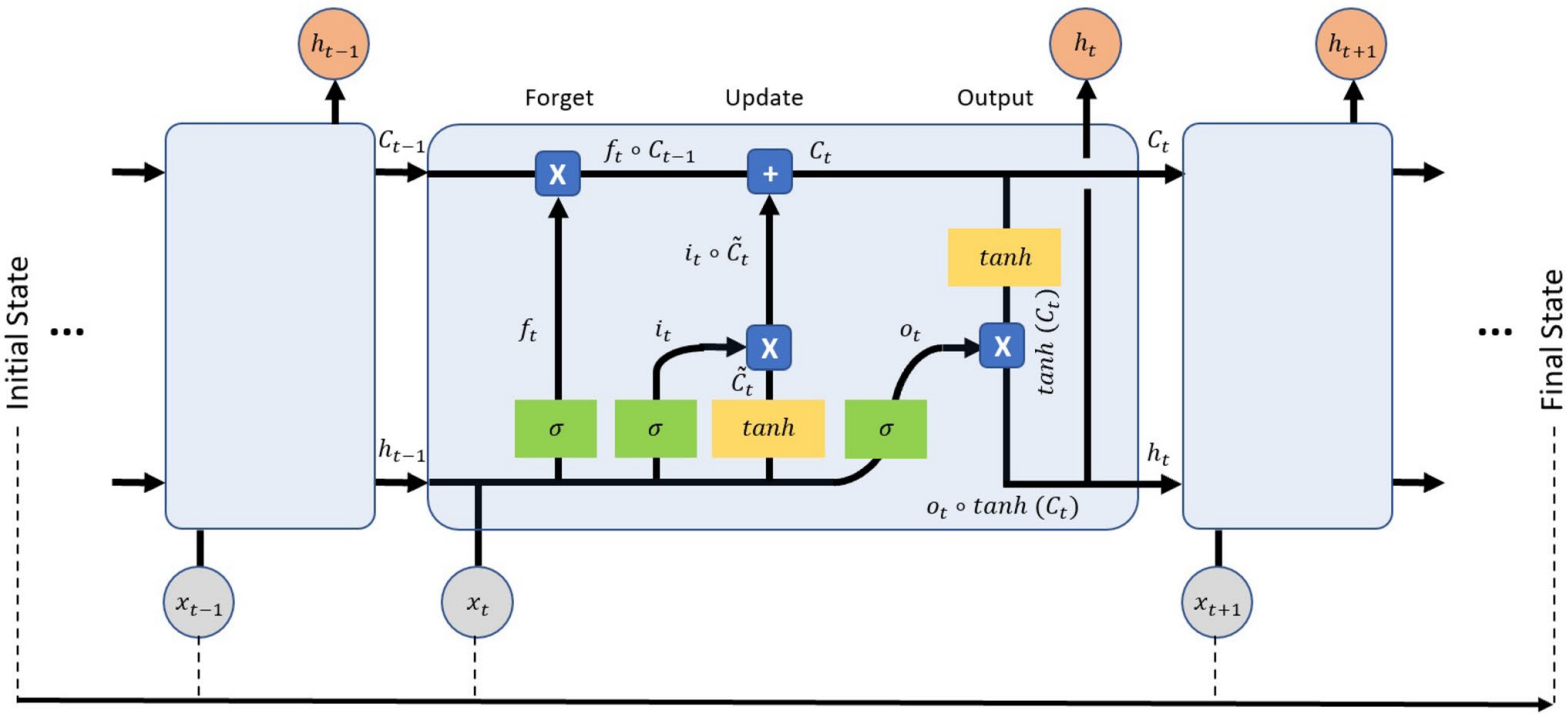
Overview of an LSTM neural processing unit. Structural characteristics of an LSTM unit and its sequence across time. $x_t$ is the input data, $h_t$ is the hidden state, $i_t$, $o_t$, and $f_t$ are gates controlling the flow of information, and $C_t$ is the cell state. The $\boxed {x}$ and $\boxed {+}$ operators represent the element-wise product ($\circ$) and summation, respectively, whereas $\sigma$ denotes the sigmoid function of $\sigma (x)=(1+e^{-x})^{-1}$.

### Long short-term memory networks

An LSTM network is a subclass of RNNs^46^, trying to circumvent RNNs’ inability to learn to recognise long-term dependencies in the data sequences. Hochreiter and Schmidhuber^74^ addressed the latter by presenting the LSTM unit, whereas LSTM networks are constructed by combining several layers of LSTM units. Figure 9 shows the structure of an LSTM unit, and its sequence across time. Each LSTM unit consists of three gates that operate on the input vector, $x_t$, to generate the cell state, $C_t$, and the hidden state, $h_t$. From a physical interpretation, the cell state can be viewed as the memory of the cell, while the gates control the flow of information in and out of the memory. In addition, the input gate determines the incorporation of new information, the forget gate determines which information should be discarded, and the output gate controls the information that passes along to the next layer. Following the interconnections presented in Fig. 9, the following formulas per category of the variables hold:

1. Gating variables:
  1 $$\begin{aligned} f_t&=\sigma (W_fx_t+U_fh_{t-1}+b_f) \end{aligned}$$
  2 $$\begin{aligned} i_t&=\sigma (W_ix_t+U_ih_{t-1}+b_i) \end{aligned}$$
  3 $$\begin{aligned} o_t&=\sigma (W_ox_t+U_oh_{t-1}+b_o) \end{aligned}$$
2. Candidate (memory) cell state variable:
  4 $$\begin{aligned} \tilde{C_t}=\tanh {(W_cx_t+U_ch_{t-1}+b_c)} \end{aligned}$$
3. Cell and hidden state variables:
  5 $$\begin{aligned} C_t&=f_t\quad \circ \quad C_{t-1}+i_t\quad \circ \quad \tilde{C_t} \end{aligned}$$
  6 $$\begin{aligned} h_t&=o_t\circ \tanh {(C_t)} \end{aligned}$$

where {*W*, *U*} and *b* are the learnable weights and bias of the LSTM layer, respectively, for the input and the recurrent connections for the input/output/forget gates and cell state; $\circ$ is the element-wise product of two vectors; $\sigma$ is a sigmoid function given by $\sigma (x)=(1+e^{-x})^{-1}$ to compute the gate activation function, whereas the hyperbolic tangent function (tanh) is used to compute the state activation function.

**Figure 10:**
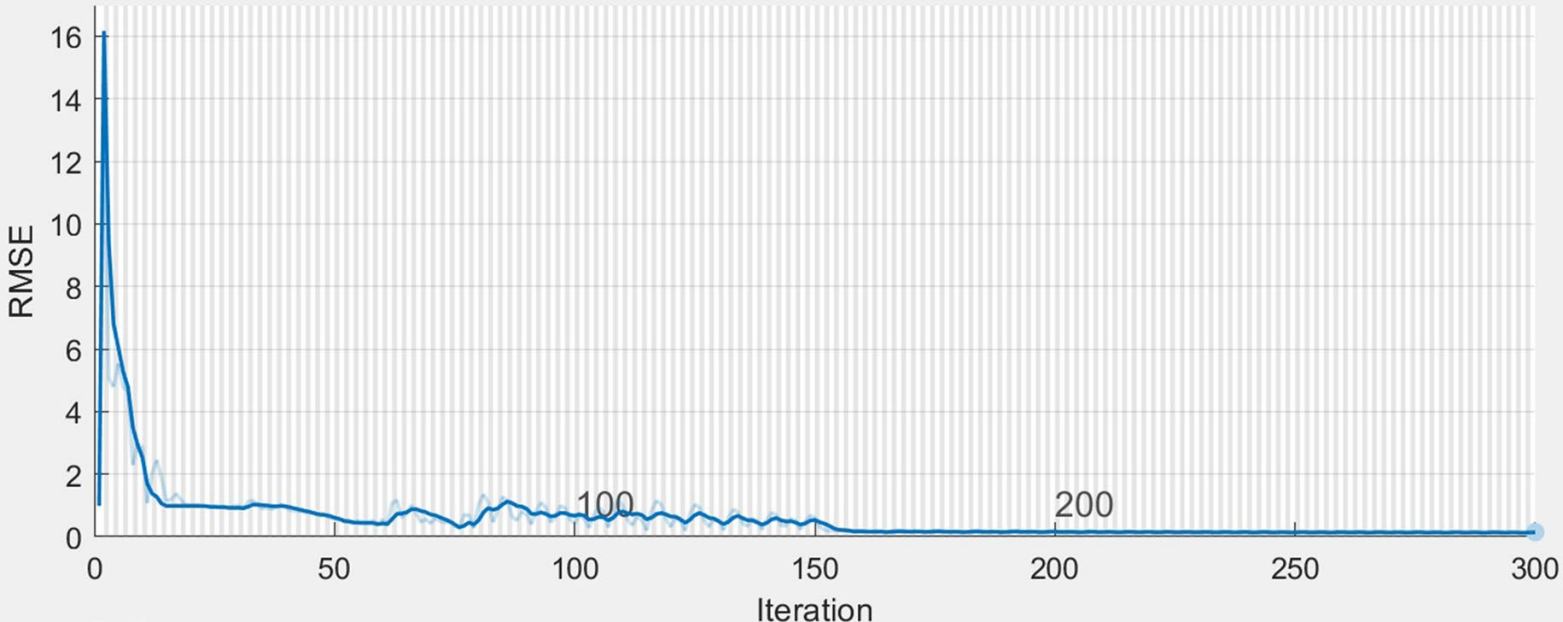
The estimated training RMSE across iterations. An example of the convergence of the estimated training RMSE to values $<0.001$ across the 300 iterations, when training the proposed LSTM network with data from DB1. The light and thick lines denote the actual and the smoothed values of the estimated training RMSE, respectively.

### Implementation issues

The final network was implemented in Matlab 2020a (The Mathworks, Natick, USA), and trained using the Adaptive Moment Estimation (Adam) optimizer^75^. The final selection of the hyperparameters of the network was based on the results from early test runs with different settings; the one which provided most promising predictive performance was finally chosen. In particular, the final network consisted of four layers, i.e., the sequence input layer, the LSTM layer with 1200 hidden units, the fully connected layer and the regression output layer, and was trained for 300 epochs. With this selection, the estimated training RMSE was converging to values less than 0.001 across the 300 iterations (Fig. 10). To prevent the gradients from exploding, the gradient threshold was set to 1. The initial learn rate was set to 0.005 and the learn rate was dropped after 150 epochs by multiplying the initial rate by a factor of 0.2. The size of the mini-batch used for each training iteration to evaluate the gradient of the loss function and update the weights was set equal to 128.

A common issue that should be considered during training any kind of neural network is overfitting, due to the highly flexible nature of the network. In order to reduce the negative effects of overfitting, apart from the dropout process described above, regularisation techniques can also be applied to reduce the generalization error. In this vein, the $L^2$ norm regularization was also adopted here^76^. This technique, also known as Tikhonov regularization and ridge regression in statistics, is a specific way of regularizing a cost function with the addition of a complexity-representing term. In the case of $L^2$ regularization in neural networks, the term is simply the squared Euclidean norm of the weight matrix of the hidden layer of the network. $L^2$ regularization usually results in much smaller weights across the entire model. An additional parameter, $\lambda$, is added to allow control of the strength of the regularization; here the value of $\lambda =0.0005$ was used.

### Training and testing

The model was trained and tested on a High Performance Computing infrastructure at KUST, Abu Dhabi, UAE (equipped with 84 Nodes, 168 Processors, 2016 Cores, 21.5 TB Mem, 23+ TB NFS), using 24 Ivy Bridge processing nodes (2x Intel Xeon E5-2697 v2, 12Core 2.7GHz, 256 GB Memory/300 GB Local storage), running in parallel. Training was realized using the first 90% of the QoI sequence per user, whereas testing was applied on its last 10%. At each time step of the input sequence, the LSTM network learnt to predict the value of the next time step (see Fig.1).

## Supplementary information

Supplementary Table S1.

## Data Availability

All data generated and analysed during this work are available from https://github.com/LeontiosH/DeepLMS/tree/DeepLMS-data.

## Code availability

All codes used in this work are available from https://github.com/LeontiosH/DeepLMS/tree/Matlab-code.
