# Supplementary material for: DeepLMS: a deep learning predictive model for supporting online learning in the Covid-19 era
Source: Sci Rep. 2020 Nov 16;10:19888. doi: 10.1038/s41598-020-76740-9 (PMC7669866; doi:10.1038/s41598-020-76740-9)
Supplement: Supplementary file 1 — Supplementary Table S1. [file 41598_2020_76740_MOESM1_ESM.pdf]

DeepLMS: A Deep Learning predictive model for supporting online learning in the Covid-19 era

Sofia B. Dias*[1], Sofia J. Hadjileontiadou*[2], José Diniz[1], and Leontios J. Hadjileontiadis[3,4,5,]**

[1]CIPER, Faculdade de Motricidade Humana, Universidade de Lisboa, Lisbon, Portugal.
[2]Department of Primary Education, Democritus University of Thrace, Alexandroupolis, Greece
[3]Department of Electrical Engineering and Computer Science, Khalifa University of Science and Technology, Abu Dhabi, UAE.
[4]Healthcare Engineering Innovation Center, Department of Biomedical Engineering, Khalifa University of Science and Technology, Abu Dhabi, UAE
[5]Department of Electrical and Computer Engineering, Aristotle University of Thessaloniki, Thessaloniki, Greece.
*These authors contributed equally to this work.
**Correspondence and requests for materials should be addressed to L.H. (email: leontios.hadjileontiadis@ku.ac.ae)

ABSTRACT

Coronavirus (Covid-19) pandemic has imposed a complete shut-down of face-to-face teaching to universities and schools, forcing a crash course for online learning plans and technology for students and faculty. In the midst of this unprecedented crisis, video conferencing platforms (e.g., Zoom, WebEx, MS Teams) and learning management systems (LMSs), like Moodle, Blackboard and Google Classroom, are being adopted and heavily used as online learning environments (OLEs). However, as such media solely provide the platform for e-interaction, effective methods that can predict the learner's behavior in the OLEs should be made available as supportive tools to educators and metacognitive triggers to learners. Here we show, for the first time, that Deep Learning techniques can be used to handle LMS users' interaction data and form a novel predictive model, namely DeepLMS, that can forecast their quality of interaction (QoI) with LMS. Using Long Short-Term Memory (LSTM) networks, DeepLMS results in average testing Root Mean Square Error (RMSE)<0.009, and average correlation coefficient between ground truth and predicted QoI values $r \geq 0.97$ ($p < 0.05$), when tested on QoI data from one database pre- and two ones during-Covid-19 pandemic. DeepLMS personalized QoI forecasting scaffolds user's online learning engagement and provides educators with an evaluation path, additionally to the content-related assessment, enriching overall view on learner motivation and participation in the learning process.

FIS#	LMS Moodle metrics ($M_1 - M_{110}$)				Categorized Parameters ($C_1 - C_{14}$)	
	$M_{\#}$	'Action'	Combination of 'Module' and 'Action'		$C_{\#}$	FIS Input Name
			'Module'	'Action'		
1	1	View all			4	Course Page (CP)
	2	View discussion			2	Forum/Discussion/Chat (F/D/C)
	3	View form			1	Journal/Wiki/Blog/Form (J/W/B/F)
	4	View forum(s)			2	Forum/Discussion/Chat (F/D/C)
	5	View grade			3	Submission/Report/Quiz/Feedback (S/R/Q/F)
	6	View graph			3	Submission/Report/Quiz/Feedback (S/R/Q/F)
	7	View report			3	Submission/Report/Quiz/Feedback (S/R/Q/F)
	8	View responses			2	Forum/Discussion/Chat (F/D/C)
	9	View submission			3	Submission/Report/Quiz/Feedback (S/R/Q/F)
	10	View subscribers			1	Journal/Wiki/Blog/Form (J/W/B/F)
	11	Preview			4	Course Page (CP)
	12	Info			1	Journal/Wiki/Blog/Form (J/W/B/F)
	13	Links			1	Journal/Wiki/Blog/Form (J/W/B/F)
	14	Search			4	Course Page (CP)
	15	Mail error			2	Forum/Discussion/Chat (F/D/C)
	16	Mark read			2	Forum/Discussion/Chat (F/D/C)
	17	Templates view			4	Course Page (CP)
	18		Course	View	4	Course Page (CP)
	19		Glossary	View	1	Journal/Wiki/Blog/Form (J/W/B/F)
	20		Resource	View	4	Course Page (CP)
	21		Assignment	View	3	Submission/Report/Quiz/Feedback (S/R/Q/F)
	22		Feedback	View	3	Submission/Report/Quiz/Feedback (S/R/Q/F)
	23		User	View	4	Course Page (CP)
	24		Choice	View	3	Submission/Report/Quiz/Feedback (S/R/Q/F)
	25		Wiki	View	1	Journal/Wiki/Blog/Form (J/W/B/F)
	26		Lesson	View	1	Journal/Wiki/Blog/Form (J/W/B/F)
	27		Blog	View	1	Journal/Wiki/Blog/Form (J/W/B/F)
	28		Notes	View	1	Journal/Wiki/Blog/Form (J/W/B/F)
	29		Quiz	View	3	Submission/Report/Quiz/Feedback (S/R/Q/F)
	30		Scorm	View	3	Submission/Report/Quiz/Feedback (S/R/Q/F)
	31		Data	View	4	Course Page (CP)
	32		Flashchat	View	2	Forum/Discussion/Chat (F/D/C)
	33		Hotpot	View	3	Submission/Report/Quiz/Feedback (S/R/Q/F)
	34		Chat	View	2	Forum/Discussion/Chat (F/D/C)
	35		Journal	View	1	Journal/Wiki/Blog/Form (J/W/B/F)

FIS#	LMS Moodle metrics ($M_1 - M_{110}$)				Categorized Parameters ($C_1 - C_{14}$)	
	$M_\#$	'Action'	Combination of 'Module' and 'Action'		$C_\#$	FIS Input Name
			'Module'	'Action'		
2	36	Add mod			5	Module (M)
	37	Add post			6	Post/Activity (P/A)
	38	Add entry			7	Resource/Assignment (R/A)
	39	Add discussion			6	Post/Activity (P/A)
	40	Add comment			6	Post/Activity (P/A)
	41	Choose			6	Post/Activity (P/A)
	42	Choose again			6	Post/Activity (P/A)
	43	Start			5	Module (M)
	44	Start complete			5	Module (M)
	45	End			5	Module (M)
	46	Fields add			5	Module (M)
	47	Report			6	Post/Activity (P/A)
	48	Report live			6	Post/Activity (P/A)
	49	Report log			6	Post/Activity (P/A)
	50	Report outline			6	Post/Activity (P/A)
	51	Report participation			6	Post/Activity (P/A)
	52	Report stats			6	Post/Activity (P/A)
	53	Submit			6	Post/Activity (P/A)
	54	Subscribe			6	Post/Activity (P/A)
	55	Subscribe all			6	Post/Activity (P/A)
	56	Talk			6	Post/Activity (P/A)
	57	Attempt			6	Post/Activity (P/A)
	58	Continue attempt			6	Post/Activity (P/A)
	59	User report			6	Post/Activity (P/A)
	60	Bogus			6	Post/Activity (P/A)
	61	Set page flags			6	Post/Activity (P/A)
	62		Resource	Add	7	Resource/Assignment (R/A)
	63		Chat	Add	6	Post/Activity (P/A)
	64		Forum	Add	6	Post/Activity (P/A)
	65		Label	Add	8	Label (L)
	66		Assignment	Add	6	Post/Activity (P/A)
	67		Feedback	Add	6	Post/Activity (P/A)
	68		Choice	Add	6	Post/Activity (P/A)
	69		Calendar	Add	6	Post/Activity (P/A)
	70		Wiki	Add	6	Post/Activity (P/A)
	71		Glossary	Add	6	Post/Activity (P/A)
	72		Data	Add	5	Module (M)
	73		Lesson	Add	5	Module (M)
	74		Flashchat	Add	6	Post/Activity (P/A)
	75		Hotpot	Add	6	Post/Activity (P/A)
	76		Journal	Add	6	Post/Activity (P/A)
	77		Quiz	Add	6	Post/Activity (P/A)
	78		Scorm	Add	5	Module (M)

FIS#	LMS Moodle metrics ($M_1 - M_{110}$)				Categorized Parameters ($C_1 - C_{14}$)	
	$M_{\#}$	'Action'	Combination of 'Module' and 'Action'		$C_{\#}$	FIS Input Name
			'Module'	'Action'		
	79		Survey	Add	5	Module (M)
3	80	Assign			11	Assign (A)
	81	Unassign			12	Edit/Delete (E/D)
	82	Enrol			11	Assign (A)
	83	Unenrol			12	Edit/Delete (E/D)
	84	Unsubscribe			12	Edit/Delete (E/D)
	85	Upload			9	Upload (UP)
	86	Attachment			9	Upload (UP)
	87	Update			10	Update (U)
	88	Update comment			10	Update (U)
	89	Update entry			10	Update (U)
	90	Update feedback			10	Update (U)
	91	Update grades			10	Update (U)
	92	Update mod			10	Update (U)
	93	Update post			10	Update (U)
	94	Fields update			10	Update (U)
	95	Recent			10	Update (U)
	96	Move discussions			10	Update (U)
	97	Templates saved			9	Upload (UP)
	98	Edit			12	Edit/Delete (E/D)
	99	Edit section			12	Edit/Delete (E/D)
	100	Edit questions			12	Edit/Delete (E/D)
	101	Delete			12	Edit/Delete (E/D)
	102	Delete all			12	Edit/Delete (E/D)
	103	Delete attempt			12	Edit/Delete (E/D)
	104	Delete comment			12	Edit/Delete (E/D)
	105	Delete discussion			12	Edit/Delete (E/D)
	106	Delete entry			12	Edit/Delete (E/D)
	107	Delete mod			12	Edit/Delete (E/D)
	108	Delete post			12	Edit/Delete (E/D)
	109	Change password			12	Edit/Delete (E/D)
	110	Review			12	Edit/Delete (E/D)
5	-	(directly from Moodle date log)			13	Time Period (TP)
	-	(directly from Moodle login/logout log)			14	Engagement Time (ET)

Table S1. Codification of all LMS Moodle metrics ($M_1 - M_{110}$) as categorized parameters ($C_1 - C_{14}$), fed as inputs to the Fuzzy Inference Systems (FIS1, FIS2, FIS3, and FS5) of the FuzzyQoI model[42]. The metrics ($M_1 - M_{110}$) sustain their original code name as exported by Moodle; the parameters of the 14 categories ($C_1 - C_{14}$) are named here as used in[42].
